# PRONTO-TK: a user-friendly PROtein Neural neTwOrk tool-kit for accessible protein function prediction

**DOI:** 10.1093/nargab/lqae112

**Published:** 2024-08-27

**Authors:** Gianfranco Politano, Alfredo Benso, Hafeez Ur Rehman, Angela Re

**Affiliations:** Department of Control and Computer Engineering, Politecnico di Torino, Torino, 10129, Italy; Department of Control and Computer Engineering, Politecnico di Torino, Torino, 10129, Italy; School of Computing and Data Sciences, Oryx Universal College with Liverpool John Moores University, Qatar; Department of Applied Science and Technology, Politecnico di Torino,Torino, 10129, Italy

## Abstract

Associating one or more Gene Ontology (GO) terms to a protein means making a statement about a particular functional characteristic of the protein. This association provides scientists with a snapshot of the biological context of the protein activity. This paper introduces PRONTO-TK, a Python-based software toolkit designed to democratize access to Neural-Network based complex protein function prediction workflows. PRONTO-TK is a user-friendly graphical interface (GUI) for empowering researchers, even those with minimal programming experience, to leverage state-of-the-art Deep Learning architectures for protein function annotation using GO terms. We demonstrate PRONTO-TK’s effectiveness on a running example, by showing how its intuitive configuration allows it to easily generate complex analyses while avoiding the complexities of building such a pipeline from scratch.

## Introduction

Understanding the functional characteristics of proteins is vital for progressing toward a better understanding of metabolic processes in living organisms. Among several classification schemes such as the Enzyme Commission (EC) numbers (1) and the Kyoto Encyclopedia of Genes and Genomes (KEGG) (2), the Gene Ontology (GO) knowledgebase devised a standardized vocabulary to describe proteins functionalities in a human- and machine-readable manner using hierarchically related functional classes organized into three different ontologies: MFO (Molecular Function Ontology), BPO (Biological Process Ontology), and CCO (Cellular Component Ontology), i.e. the functional role(s), biological mechanism(s), and cellular component(s) or sub-cellular localization(s) in which the protein acts (3). GO terms can facilitate the annotation and classification of gene products in the growing number of genome sequences and molecular profiling datasets, accelerating our understanding of individual species’ and complex ecosystems’ functioning (4,5), and enabling the conception of metabolic engineering and synthetic biology tools for basic and applied biological research (6). Since designing and running experiments to functionally annotate proteins is expensive and time-consuming, most proteins currently without known functions (i.e. assigned GO terms) are unlikely to be experimentally evaluated anytime soon. Breakthroughs in natural Language Models (LM) (7) have recently shown their enormous potential in filling the sequence-annotation gap by generating descriptive representations, i.e. embeddings, for the steadily growing number of proteins with unknown function from just their sequences (8–11) and overcoming the limitations of standard feature-based Machine Learning (ML) methods. LM representations have been used as input to subsequent supervised training (12), thereby transferring knowledge acquired during task-agnostic, self-supervised pre-training on massive amounts of proteins to the task of predicting specific aspects of protein functionality. Existing studies showed the embeddings-based approach features remarkable predictive performance (13,14) on various tasks relevant for computational biology such as shedding light on gene regulation (15) by predicting, among others, protein–nucleotide interfaces (16,17), chromatin accessibility (18) and phenotypes (19). Despite the success of Deep Learning applications in recent years, a key challenge remains: making these powerful tools accessible to life science researchers who may not have extensive programming experience.

This paper introduces PRONTO-TK, an open-source Python PROtein Neural neTwOrk ToolKit, designed to address this gap. PRONTO-TK simplifies the use of two-stage transfer learning. This approach leverages cutting-edge, LM-based representations of biological sequences, as described in (20), to generate predictions for protein function across different species, using GO terms. To translate proteins into embeddings, PRONTO-TK utilizes the pre-trained model ProtT5-XL, a self-supervised model that was shown, in a recent study (20), to outperform existing methods relying on evolutionary information. Its effectiveness in two-stage transfer learning for complex prediction tasks has been further confirmed in (21). As the main source of data, PRONTO-TK can download datasets from the UniProt database (22). PRONTO-TK is an open-source project and can be downloaded from https://github.com/alfredobenso/PRONTO-TK and https://doi.org/10.5281/zenodo.13272726.

PRONTO-TK is a user-friendly solution that streamlines the prediction pipeline, from dataset creation, to training and test, and to final validation. In particular, it has been designed to perform the following tasks:

- to download from UniProt, given one main GO term and a set of taxonomic identifiers, a dataset of proteins annotated with the required GO term (and possibly its descendants), as well as a dataset of proteins NOT annotated with the same term(s).
- to process the protein sequence dataset and convert its protein sequences into embeddings.
- to train, test and fine-tune Deep Learning models using the selected datasets, while allowing users to explore different combinations of hyper-parameters. In this phase, the configuration file allows to merge together more dataset files.
- to validate/infer the functionality of unknown (during training) protein sequences, providing each protein with a probability of being tagged with the initial GO term (but not with descendant GO terms, since multi-label predictions would require a different setup).

In the realm of protein function prediction, PRONTO-TK stands out for focusing primarily on single GO term annotation, which enhances prediction accuracy by leveraging state-of-the-art Deep Learning models. This singular focus allows PRONTO-TK optimizing predictions for specific protein functions, and differentiates itself from traditional multi-label prediction tools like NetGO 3.0 (11), DeepGOWeb (23), TALE (24) and ATGO (25). Its flexibility in hyper-parameter exploration during model training further enhances customization options, distinguishing it from tools like PANNZER (26) and PANDA (27) (28). PRONTO-TK also excels in comprehensive validation, evaluating the probability of GO term predictions for individual protein sequences, thus ensuring robust prediction reliability. Addressing the computational demands of large datasets efficiently, PRONTO-TK can support researchers in advanced protein function predictions.

In the remainder of this paper we will use a running example to demonstrate the PRONTO-TK main features and flexibility. We will create a pipeline able to annotate proteins with the GO term ‘DNA binding transcription factor activity (GO:0003700)’. We chose this problem because Transcription Factors (TFs) are sequence-specific DNA-binding proteins that exert a central role in transcriptional initiation. TFs promote or block the RNA polymerase to regulate the rates of the transcription of a set of genes. Analyzing transcriptional regulation enables us to understand an essential regulatory layer by which an organism controls the expression of genes in response to genetic or environmental cues. Identification of TFs is a starting point for elucidating transcriptional regulatory activity.

The reader should also keep in mind that the goal of this paper and of this example is to demonstrate the flexibility of the PRONTO-TK’s pipeline in fast prototyping and testing existing or future Deep Learning models that operate on protein embeddings, and not to propose a novel annotation algorithm.

## Materials and methods

PRONTO-TK allows defining two separate pipelines: a Data Pipeline to create an embeddings’ dataset from UniProt queries, and a Training/Test/Validation one to run a Deep Learning experiment on those datasets. Both pipelines are configured through a configuration file that allows controlling all the functionalities of the tool. The use of a configuration file serves as a user-friendly alternative to writing complex code for defining pipeline parameters. No advanced Python programming skills are necessary to use PRONTO-TK.

**Figure 1.**
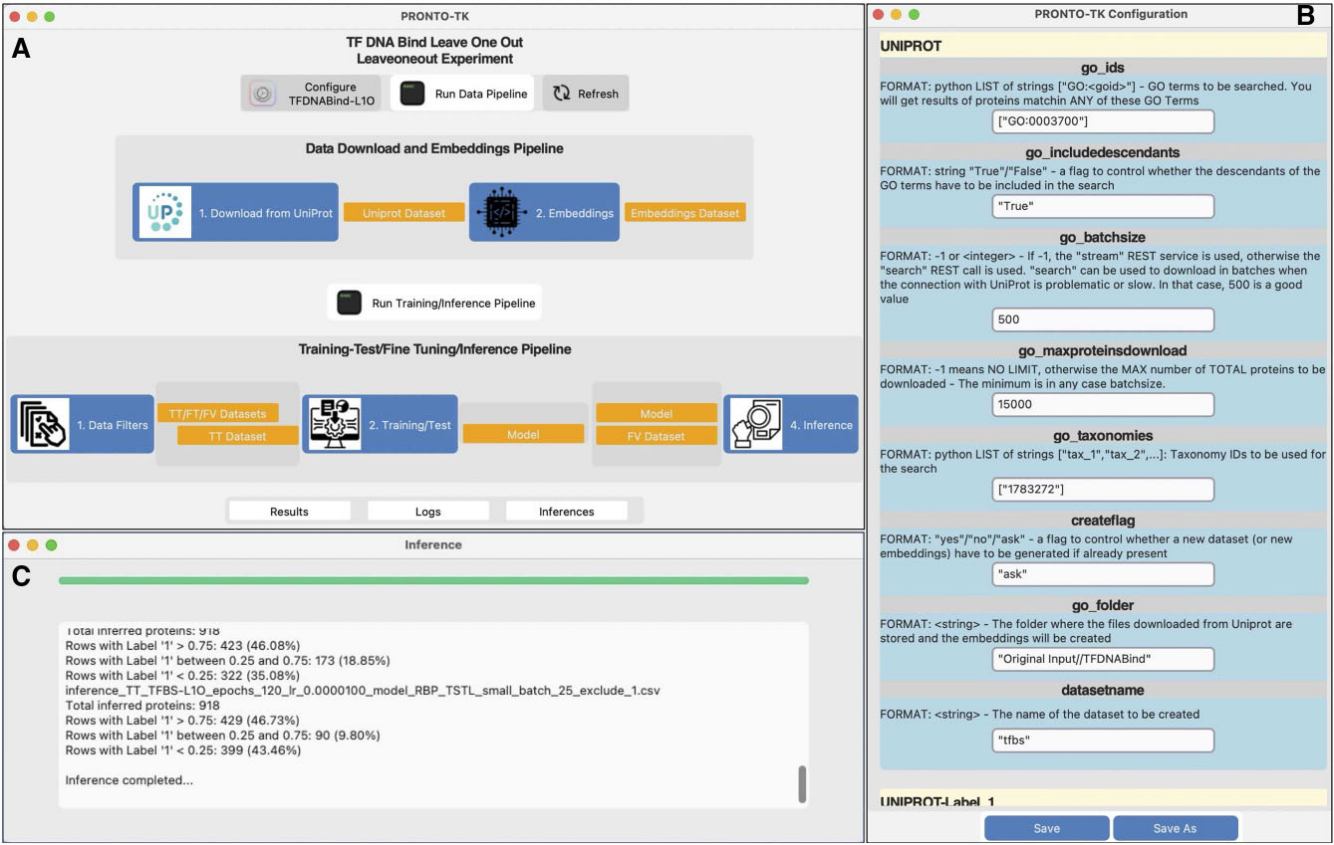
PRONTO-TK’s Graphical User Interface. (**A**) Main Control Panel: each pipeline can be enabled in full (by clicking on the run button), or step-by-step (by clicking on each individual phase). Each phase is enabled if the correct input files are present. Available files are colored in orange, whereas missing ones are colored in purple. If only some of the required files are present, the file box is colored in light gray. (**B**) Configuration Panel for the experiments’ configuration files. The configuration panel allows setting all the configuration parameters. A short help precedes every configuration item. (**C**) Log window for each step of the pipeline. A progress bar and a text box allow to track the task progress.

PRONTO-TK’s GUI is presented in Figure 1 and it allows users to both run each pipeline in batch, or one phase at a time. Clicking each phase opens a separate window that includes a progress bar and a very detailed real-time log of the executed operations. The Data Pipeline consists of two separate phases:

1. **Download from UniProt**: the first step of the Data Pipeline downloads from UniProt a dataset of proteins annotated with a set of user-selected GO term (the main one and possibly its descendants). The configuration file let users decide if to look for proteins annotated with the chosen GO term only, or the ones tagged with the chosen GO term and any of its descendants in the Gene Ontology. The tool envisages the option of downloading the whole proteins associated with a certain GO term that include the proteins directly associated with the GO term and the proteins directly associated with any of its descendants. With this step, we prepare the data that will be later used to train the Deep Learning models. The configuration file allows defining how to select proteins that will be Labeled as ’1’ (i.e. labelled with the chosen GO term(s)) and how to select the Label ’0’ proteins. Besides the target GO terms, the configuration file allows to select the Annotation Type, i.e. the evidence upon which a GO annotation is based on (*manual*, *automatic*, or both), the Review flag indicating whether the selected proteins belong to the Swiss-Prot section of UniProtKB (*reviewed*) or to the computer-annotated TrEMBL section (*unreviewed*), and a maximum number of records to download. UniProt is not the only possible source of data. Any other source of data can be used, provided that the format of the data set is identical to the one provided by UniProt (in terms of labels and type of information). In our illustrative experiment, we chose to download from UniProt the proteins associated with the GO:0003700 term (DNA-binding transcription factor activity) and **any of** its descendants, and to select proteins from the Terrabacteria taxonomy (#1783272). We configured the tool to download all proteins that are annotated with the selected **GO terms** assigned the *reviewed* status, regardless of the assertion mode (*manual* or *automatic*). To select the ‘Label 0 proteins’ (i.e. proteins not annotated with any of the selected GO terms) we chose *reviewed* proteins provided with *manual* annotation. When executed, this task downloaded a pool of 2557 proteins to be used in the experiment.
2. **Protein embedding**: before being able to be used by a Neural Network, each protein sequence is transformed into a set of 1024 embeddings using (https://github.com/agemagician/ProtTrans). A state-of-the-art Deep Learning model (*prot-t5-xl-half-uniref50-enc* was the best model allowed by our hardware, but it can be easily changed in the configuration if a more powerful hardware is available. The final embeddings dataset size is, in our example, 336 MB. Using embeddings to represent protein sequences mitigates concerns related to sequence identity, as they capture higher-level functional and structural properties beyond simple sequence similarity. This approach ensures robust model performance evaluation, making traditional sequence identity-based data splitting less critical.

**Figure 2.**
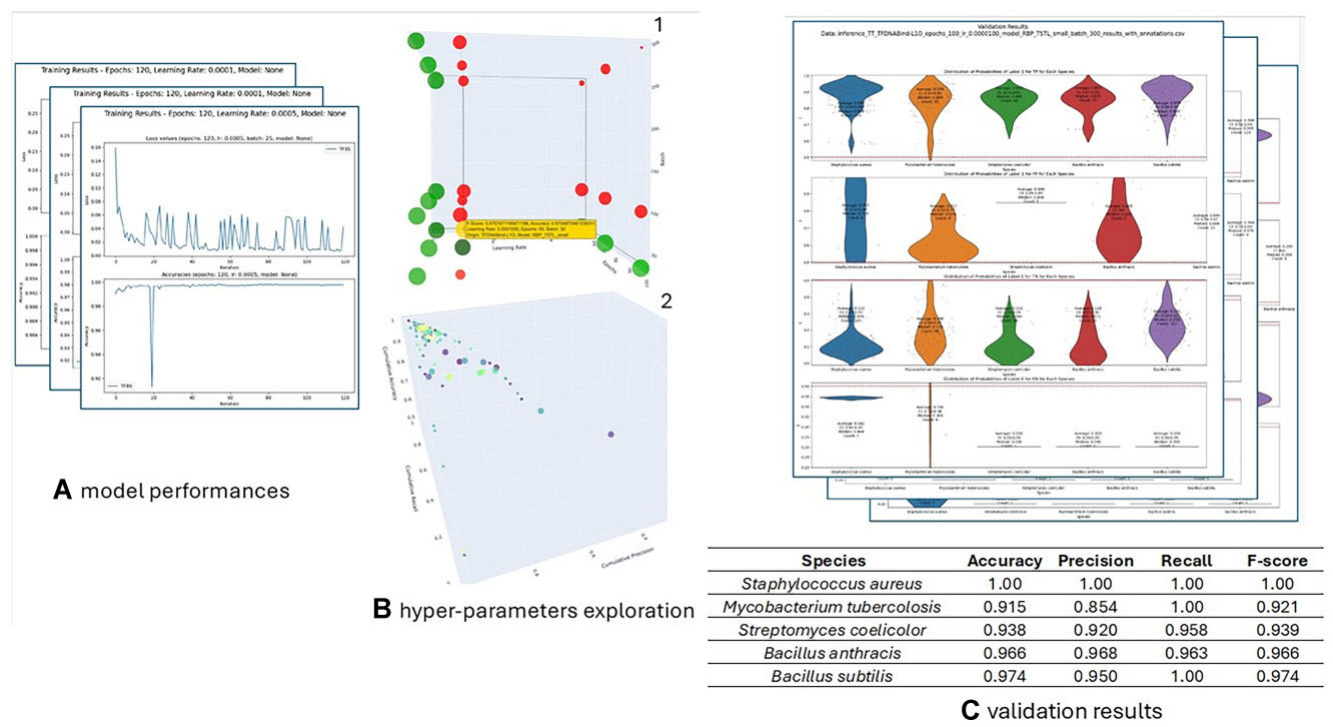
PRONTO-TK’s visual outputs. (**A**). A plot of loss and accuracy versus epochs for each trained or fine-tuned model. (**B-1**). 3D plot summarizing the performances of the trained models. Each point corresponds to a combination of the input parameters (in this case we have 27 points corresponding to the 27 models). The color of the dot derives from the F-score of the model. Red dots have an *F*-score < 0.9. Green dots are shaded accordingly to their F-score (darker green is a lower F-score). The yellow dot corresponds to the model with the best *F*-score. Moving the mouse over a dot shows its parameters. (**B-2**) 3D plot of Accuracy, Precision, and Recall for all trained models. Larger points correspond to the average values (of Accuracy, Precision, and Recall) for all models trained with the same parameters for each leave-one-species-out species. Moving the mouse over a point will display the corresponding model parameters. (**C**). For each combination of model parameters, the violin plots of the distribution of the predicted probabilities for each label in the validation of each ‘leave-one-species-out’ species. This is repeated for true positives (TP), false positives (FP), true negatives (TN) and false negatives (FN), followed by a table reporting the measures of the predictive performances achieved by the model for each species.

The Training/Test/Validation Pipeline implements the following steps:

1. **Data filtering**: after generating the final embeddings’ dataset, the tool allows customizing how to select data for the different phases of the pipeline (Training, Fine Tuning, and Validation/Inference). Again, the parameters are Annotation Type/Review/Species. In case of data overlapping between Training and Fine Tuning (if for example the user chooses the same filters for both phases), it is possible to specify the percentage of data to allocate to each phase. PRONTO-TK affords two different types of experiment:
  - **Single**: in this case the dataset is typically divided into two parts: a training set and a validation set. The model is trained on the training set and then evaluated on the validation set. This is a straightforward method to assess the model’s performance. The configuration allows users to exclude or include different species in each of the two steps.
  - **Leave-one-species-out**: in a Leave-One-Species-Out experiment, the model is trained multiple times. In each iteration, one species is left out from the training set and used as the validation set. This process is repeated for each configured species (or group of species). This method is particularly useful to estimate the model accuracy in classifying “de novo” species.
2. **Training, test and fine tuning**: Training, Test, and Fine Tuning are carried out using one of the two models presented in (21). Nevertheless, the code allows, theoretically, users to create and use custom models, provided their functional and data interface remains the same. The tool also allows users to easily perform hyper-parameter tuning (in both Training and Fine Tuning) by defining a range of batch sizes, epochs, and learning rates to explore. To set these parameters, in our experiment we used the following rule-of-thumb:
  \begin{equation*} 3 * DatasetSize \approx (batchSize * Epochs) \end{equation*}
  In our example, given a dataset of 2557 proteins, we decided to explore all configurations corresponding to the combination of the following parameters: batch size: 30, 100, 300; epoch: 50, 75, 100; learning rate: 0.0005, 0.0001, 0.00001 (for a total of 27 different models). At the end of the experiment PRONTO-TK produces several plots that can support the user in evaluating the best model to choose (Figure 2A, B). In our transcription factor annotation experiment we decided not to use Fine Tuning, and to select all available data for training and testing. In order not to compromise the size of the training while maintaining a statistically adequate number of proteins for validation, we selected the following leave-one-species-out species: *Bacillus Subtilis*, *Bacillus anthracis*, *Mycobacterium tuberculosis*, *Streptomyces coelicolor* and *Staphylococcus aureus*.
3. **Inference**: the tool allows running inference on both labelled and unlabelled datasets and executing a basic set of statistical analyses. For labelled datasets, it produces a final table reporting accuracy, precision, recall and *F*-score, as well as a set of plots (see Figure 2). In a leave-one-species-out experiment like the proposed one, these results make it possible to better understand and quantify how the model will behave when inferring GO terms for proteins never used in the training phase.

## Implementation

PRONTO-TK is fully implemented in Python. All experiments reported in this paper ran on a Mac Book Pro M2 with 64GB of RAM and a Metal Performance Shaders (MPS) to leverage the GPU on MacOS devices. The code is freely available on GitHub and Zenodo and its modular organization makes it easier for the community to modify it and to participate in improving the tool and extend its capabilities.

## Results

We evaluated the performance of our pipeline in predicting protein annotations with four measures: accuracy, precision, recall and the *F*-score. As anticipated, PRONTO-TK is characterized by the unusual co-existence of ease in usage and considerable room for personalized configuration of the pipeline. To illustrate this remarkable feature, we presented the outcomes of the pipeline in the selected species in relation to different hyper-parameters settings. It is worth noting that the sensitivity analysis drafted here is not meant to rigorously meet any optimization of predictive performances. Nonetheless, as shown in Figure 2C, the pipeline achieved high performance as accuracy was found to vary, across the target species for transcription factor prediction, in the range 0.91–1.0, precision in the range 0.85–1.0, recall in the range 0.96–1.0 and the *F*-score in the range 0.92–1.0.

To optimize and improve the overall classifier accuracy, we leveraged the visualization capabilities of our tool, as depicted in Figure 2B.1, to comprehensively analyze, adjust, and refine our hyper-parameter settings, namely Learning Rate (LR), Epoch Number (EN), and Batch Size (BS). Here, we provide a brief explanation about the interpretation of the plot presented in Figure 2B.

The plot clearly illustrates that LR stands out as the most critical parameter. Although an optimal solution exists at intermediate LR (LR = 0.0001), such LR setting exhibits instability, as evidenced by its considerable fluctuation in F-score in response to changes in other meta-parameters (BS and EN). However, increasing the LR, although resulting in sub-optimal solutions in terms of accuracy or F-score, leads to an overall increased stability of the solution pool, denoted by points remaining solid green despite changes in other meta-parameters. Consequently, in this example we would initially select the most reliable LR and subsequently fine-tune other parameters in the quasi-optimal solution space. This entire process may be iterated by updating meta-parameters boundaries to allow for a coarser and finer exploration of the parameters.

## Discussion and conclusions

PRONTO-TK makes protein function prediction accessible to a broad scientific audience, extending the access to state-of-the-art ML tools based on representative languages to researchers with limited programming expertise. This user-friendly toolkit has the potential to accelerate discoveries in functional genomics and protein science because it contrasts the complexity of writing code for each stage of the data pipeline (data download, pre-processing, model training, inference) with the ease of configuring these steps in PRONTO-TK’s configuration file and GUI. The fine-grained configurability allows researchers to tailor the prediction process to their specific needs and to the specific datasets. Last but not least, the PRONTO-TK’s pipeline accelerates protein function discovery and facilitates large-scale functional annotation while standardizing the process and therefore enhancing reproducibility of the results.

## Data Availability

PRONTO-TK, along with its documentation and manuals, is available at https://github.com/alfredobenso/PRONTO-TK and https://doi.org/10.5281/zenodo.13272726.
